# Transcription Factor Profiling Identifies Spatially Heterogenous Mediators of Follicular Thyroid Cancer Invasion

**DOI:** 10.1007/s12022-020-09651-0

**Published:** 2020-10-16

**Authors:** Norman G. Nicolson, Johan O. Paulsson, C. Christofer Juhlin, Tobias Carling, Reju Korah

**Affiliations:** 1 Yale Endocrine Neoplasia Laboratory, Department of Surgery, Yale School of Medicine, New Haven, CT USA; 2 Department of Oncology-Pathology, Karolinska Institutet, Solna, Sweden; 3 Department of Pathology and Cytology, Karolinska University Hospital, Stockholm, Sweden; 4 Carling Adrenal Center, Tampa, FL USA

**Keywords:** Follicular thyroid cancer, Transcription factor, Invasion, Widely invasive

## Abstract

While minimally invasive follicular thyroid cancer (miFTC) generally has low risk of recurrence or death, encapsulated angioinvasive (eaFTC) or widely invasive (wiFTC) histological subtypes display significantly worse prognosis. Drivers of invasion are incompletely understood. Therefore, tissue samples including miFTC, eaFTC, and wiFTC tumors, as well as histologically normal thyroid adjacent to benign follicular adenomas, were selected from a cohort (*n* = 21) of thyroid tumor patients, and the gene expression of selected transcription factors was characterized with quantitative PCR. Invasion-relevant spatial expression patterns of selected transcription factors were subsequently characterized with immunohistochemistry. *E2F1* was over-expressed in all 3 subtypes (*p*<0.01). *SP1* was differentially expressed in eaFTC and wiFTC compared with normal (*p*=0.01 and 0.04, respectively). *TCF7L2* was significantly upregulated in wiFTC specifically (*p*<0.05). While these findings were mRNA specific, immunohistochemistry of additional cancer-associated transcription factors revealed differential expression along the tumor invasive front relative to the central tumor, and histone acetylation modulators emerged as putative invasion markers. These findings may have significant implications for the interpretation of bulk gene expression analysis of thyroid tumor samples or for the development of targeted therapeutics for this rare but aggressive thyroid cancer variant.

**Electronic supplementary material:**

The online version of this article (10.1007/s12022-020-09651-0) contains supplementary material, which is available to authorized users.

## Introduction

Follicular thyroid cancer (FTC) is the second most common type of well-differentiated thyroid cancer, making up about 10–15% of thyroid cancer diagnoses overall [1, 2]. The prognosis is generally quite favorable, especially for minimally invasive FTC (miFTC), for which recurrences or disease-specific deaths are quite rare [3, 4]. In 2017, the World Health Organization released updated histopathological guidelines for FTC, which divided the disease into minimally invasive (miFTC), encapsulated angioinvasive (eaFTC), and widely invasive (wiFTC) subtypes [5]. Unfortunately, definitively distinguishing these categories from one another, and even distinguishing benign follicular adenoma from FTC, requires a complete surgical specimen to assess the degree of capsular or vascular invasion, which cannot readily be determined from fine-needle aspiration (FNA) cytology specimens [6].

In light of this limitation, patients with indeterminate cytological findings after FNA are often faced with undergoing so-called diagnostic surgery (usually hemithyroidectomy) to arrive at a final diagnosis, which might in many cases require a return to the operating room for definitive surgery [7]. To avoid this situation, there has been intense interest in using molecular markers, such as gene expression, mutation analysis, micro-RNA profiling, or other techniques, to improve the pre-operative evaluation of indeterminate thyroid nodules [8, 9]. This has been somewhat successful for papillary thyroid cancer (PTC), the most common well-differentiated thyroid cancer, but less so for FTC [10].

For example, many FTCs have been shown to carry mutations in the *KRAS*, *NRAS*, or *HRAS* genes, or *PAX8-PPARG* gene fusions, but these have also been commonly described in adenomas and therefore have unclear ability to distinguish follicular adenomas from FTCs [11–14]. Many early studies included few or no wiFTCs and therefore could not predict the degree of FTC invasion on the basis of their included molecular markers [9]. A recent study from our group profiled the genetic landscape of miFTC, eaFTC, and wiFTC; no definitive genetic predictors of invasion were identified, although mutational burden was shown to be an important predictor of prognosis independent of histological classification [4]. Moreover, recurrent mutations of the *TERT* promoter are found in subsets of FTCs with particular poor prognosis, and recent studies have suggested that this mutation might be able to discriminate between malignant and benign follicular thyroid neoplasia [15–18]. A marker of invasive FTC might serve some function in distinguishing FTC from adenoma but might also provide guidance as to the intensity of therapy or follow-up, which could be quite different for miFTC versus wiFTC.

Given the lack of genetic predictors at the DNA level for invasive FTC behavior, we next elected to investigate potential regulation of invasion-relevant gene expression on a similar cohort of FTC cases. Transcription factor profiling using a quantitative real-time PCR (qPCR) array of 84 genes was selected as a way to identify upstream mediators of purported invasion-promoting proteins. Mediators thus identified in wiFTC and eaFTC might be useful as diagnostic or prognostic biomarkers and as potential drug targets for precision therapy for these rare but aggressive FTC subtypes.

## Materials and Methods

### Cohort Selection and Human Subject Protections

The patients included in this study received surgical treatment at Yale-New Haven Hospital or Karolinska University Hospital (either thyroid lobectomy or total thyroidectomy). All samples were independently reviewed by experienced endocrine pathologists for confirmation of the diagnosis. The diagnosis of FTC and degree of invasion were determined according to the 2017 WHO guidelines [5]. Samples were snap-frozen in liquid nitrogen (for RNA analysis) and/or fixed in formalin and paraffin-embedded (for immunohistochemistry). In total, we collected fresh-frozen tissue from 17 FTC specimen (8 miFTCs, 5 eaFTCs, and 4 wiFTCs) and 4 normal thyroid samples (grossly normal thyroid tissue derived from tissue adjacent to resected follicular thyroid adenomas) for targeted transcriptome analyses and also included a total of 30 FTC specimen available as formalin-fixed paraffin-embedded (FFPE) tissue for immunohistochemical verifications (7 miFTCs, 5 eaFTCs, and 18 wiFTCs). As additional controls, an FFPE series of 10 follicular thyroid adenomas (FTAs) were also included. Informed consent was obtained from all involved patients. Patient anonymity was protected as specified by the Health Insurance Portability and Accountability Act (Yale) and Swedish Biobank laws (Karolinska). The study was approved by the Yale University Institutional Review Board and the Swedish Ethical Review Authority.

### Gene Expression Array by Quantitative Reverse-Transcriptase PCR

A targeted transcriptome analysis was performed to examine the expression of 84 transcription factors in FTC (Supplementary Table 1). Samples from miFTC (*n* = 8), eaFTC (*n* = 5), and wiFTC (*n* = 4), as well as normal thyroid controls (*n* = 4), were tested to determine if gene expression was correlated with invasive status. Control samples were obtained from histologically normal thyroid tissue adjacent to follicular thyroid adenomas, as no truly normal thyroids were available snap-frozen in either tissue bank.

**Table 1:** Fold change transcription factor gene expression values in follicular thyroid carcinoma

Gene	miFTC	eaFTC	wiFTC
JUN	0.18	0.04	0.12
HAND2	0.15	0.17	0.07
EGR1	0.29	0.03	1.29
FOS	0.36	0.05	0.67
FOXO1	0.36	0.25	0.17
JUNB	0.29	0.19	0.77
NFATC2	0.44	0.35	0.28
REL	0.62	0.30	0.21
NFATC1	0.37	0.60	0.45
SP1	0.62	0.40	0.33
IRF1	0.59	0.46	0.45
NFATC3	0.88	0.37	0.41
STAT3	0.70	0.83	0.33
DR1	0.81	0.70	0.40
TFAP2A	0.86	0.58	0.47
SP3	0.91	0.71	0.39
CREBBP	0.93	0.72	0.51
NFAT5	1.08	0.68	0.49
STAT5A	1.00	1.05	0.53
TCF7L2	0.93	0.70	2.28
RELA	1.14	1.29	1.39
ATF1	1.24	1.55	1.08
MAX	1.36	1.15	1.47
HSF1	1.19	1.39	1.57
MEF2A	1.16	1.46	1.70
GTF2F1	1.34	1.20	1.96
HIF1A	1.37	1.84	1.48
YY1	1.38	1.76	1.61
RB1	1.46	2.32	1.20
E2F1	5.34	11.30	8.36

*miFTC* minimally invasive follicular thyroid carcinoma, *eaFTC* encapsulated angioinvasive follicular thyroid carcinoma, *wiFTC* widely invasive follicular thyroid carcinoma

RNA was first isolated from fresh, frozen tissue using the RNeasy Plus Mini Kit (Qiagen, MD, USA). cDNA was synthesized using the RT^2^ First-Strand Kit (Qiagen), and gene expression was determined with RT^2^ Profiler PCR Array PAHS-075Z for human transcription factors (Qiagen) in accordance with the directions of the manufacturer. Quantitative reverse-transcriptase PCR (qPCR) was carried out using a CFX96 Real-Time System thermocycler (Bio-Rad, CA, USA).

Threshold cycle values thus obtained were compared within each sample to the mean of all 84 examined transcription factor genes to avoid any confounding by changes in housekeeping gene levels across the categories, with further calculation of relative expression levels performed according to the usual Livak method [19]. Categories with fewer than 3 successful amplifications for a given gene were excluded.

### Immunohistochemistry

Formalin-fixed, paraffin-embedded samples from 20 FTCs were studied for immunohistochemical expression of the cancer-related transcription factors YY1, MAX, IRF1, and STAT6, including 3 miFTCs, 4 eaFTCs, and 13 wiFTCs. Moreover, we also screened ten additional FTCs (4 miFTCs, 1 eaFTC and 5 wiFTCs) for SP1, E2F1, TCF7L2, and HDAC1. Moreover, 10 FTAs were also stained for YY1, MAX, and HDAC1. All sections were subjected to a standardized immunohistochemistry (IHC) protocol. Briefly, 4-mm-thick FFPE tissue sections were deparaffinized in xylene and hydrated in a serial dilution of ethanol. Antigen retrieval was optimized for each antibody by incubating the slides for 5 min at 110 °C in a pressure cooker using citrate buffer or EDTA buffer. Endogenous peroxidase was blocked with hydrogen peroxidase and non-specific binding was blocked with 1% bovine serum albumin. The tissue slides were incubated with primary antibody for 30 min at room temperature or in 4 °C overnight followed by biotinylated secondary anti-rabbit IgG antibody BA-1000 (Vectorlabs, CA, USA). Sections were then incubated in Vectastain Elite ABC reagent (Vectorlabs) according to the manufacturer’s instructions and then incubated with peroxidase substrate solution ImmPACT DAB SK-4105 (Vectorlabs) and finally counterstained with hematoxylin. Each sample contained adjacent normal thyroid tissue to serve as control. In addition, breast cancer, colon cancer, and fibroblasts served as positive controls where appropriate. Non-specific binding of the secondary antibody was controlled for by omitting the primary antibody, as we lacked blocking peptides for adsorption experiments. The following primary antibodies were used: ab101271 rabbit polyclonal to MAX, ab76151 rabbit monoclonal TCF7L2, ab109228 rabbit monoclonal YY1, ab186384 rabbit monoclonal IRF1, ab32520 rabbit monoclonal STAT6, ab124804 rabbit monoclonal SP1, ab179445 rabbit monoclonal E2F1, and ab19845 rabbit polyclonal HDAC1 (all from Abcam, Cambridge, UK).

Initial protein targets for staining were chosen on the basis of differential expression in the qPCR array and/or published associations with cancer cell invasion, and a histone de-acetylase was studied after its potential significance was demonstrated in in silico protein-protein interaction. The distribution of staining was characterized both within the cell (i.e., cytoplasmic versus nuclear staining) and across the tumor and normal tissue (i.e., within the invasive front of the tumor, within the bulk of the tumor, or within the surrounding non-neoplastic tissue). Stainings were reported as negative (< 5% immunoreactive tumor cells), weak, medium, or strong (faint/medium/intense immunoreactivity in the majority of tumor cells respectively).

### Protein Interaction Analysis

Based on the protein expression results via immunostaining, in silico protein-protein interaction mapping was performed. Using the STRING tool, with “highest” confidence interactions from experimental, database, co-expression, neighborhood, and co-occurrence data, an interaction network was generated [20]. The starting gene list was composed of three genes selected based on immunohistochemical expression profile, and up to 10 first shell interactors were allowed.

### Statistical Analysis

Gene expression array results were log-transformed after being normalized to expression levels in the non-neoplastic control samples. Unpaired two-sided *t* tests were used to determine statistical significance of differences in expression between categories. Genes were considered to be differentially expressed if there was a more than twofold change in expression between categories, with a significance threshold of *p*<0.05 (not corrected for multiplicity given the exploratory nature of the analysis).

## Results

### Gene Expression Array

**Fig. 1:**
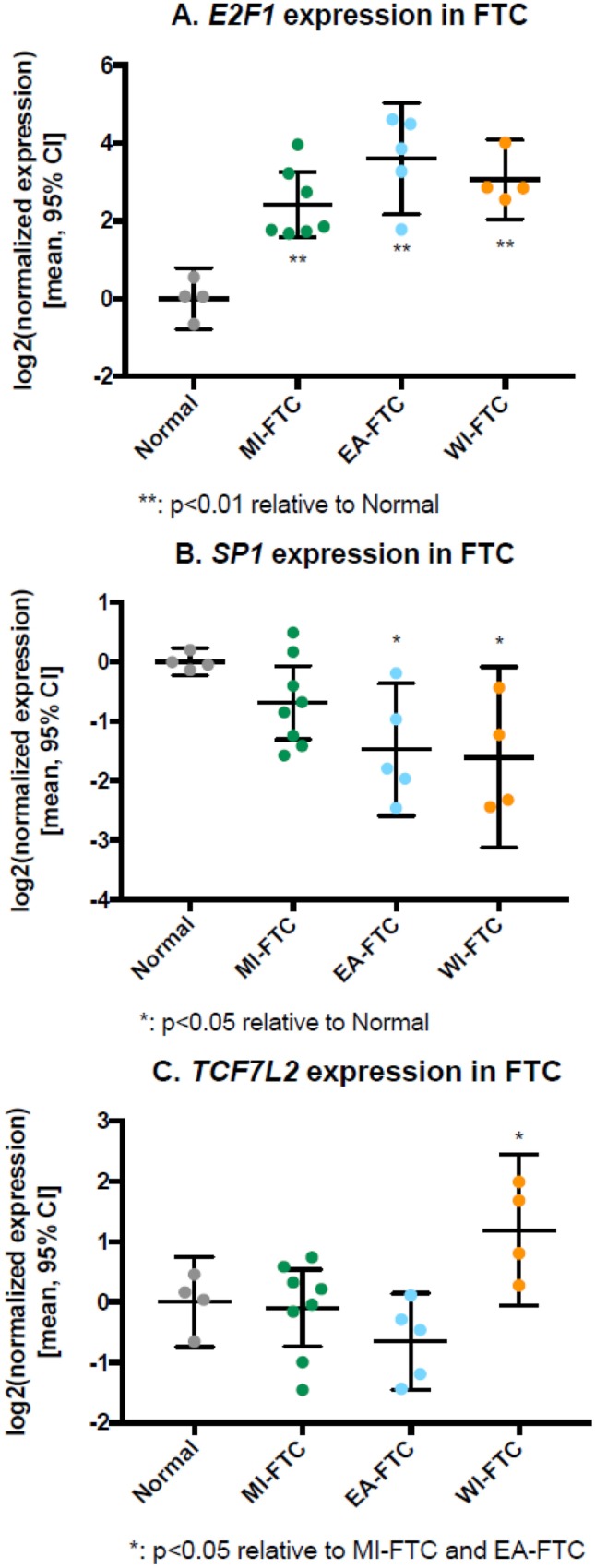
Gene expression of transcription factors *E2F1*, *SP1*, and *TCF7L2* in follicular thyroid cancers by quantitative PCR.** a** *E2F1* was over-expressed in all 3 classes of follicular thyroid cancer relative to non-neoplastic thyroid tissue (*p*<0.01). **b** *SP1* was expressed at lower levels in angioinvasive (EA-) and widely invasive (WI-) follicular thyroid carcinoma (FTC) subtypes relative to control non-neoplastic thyroid (*pcell cycle and proliferation, was overexpressed in*<0.05). Minimally invasive FTC did have lower expression than control, but the difference was not significant. **c** *TCF7L2* was expressed highly in WI-FTC specifically (*p*<0.05), while it was not significantly differently expressed in less invasive FTC subtypes

Samples from miFTC (*n* = 8), eaFTC (*n* = 5), and wiFTC (*n* = 4), as well as normal thyroid controls (*n* = 4) were profiled for their transcription factor gene expression. In total, 77 of 84 transcription factor genes were expressed in at least 3 samples per category and were included in further analysis. Of these, 22 were differentially expressed between FTC and normal or between different categories of FTC (Table 1). E2F transcription factor 1 (*E2F1*), a ubiquitous transcription factor associated with cell cycle and proliferation, was over-expressed in all 3 FTC subtypes relative to non-neoplastic thyroid (*p*<0.01; Fig. 1a). Specificity factor 1 (*SP1*), previously shown to modulate invasion in breast, prostate, and gastric cancers, was differentially expressed in eaFTC and wiFTC compared with normal (*p*<0.05; Fig. 1b). Transcription factor 7-like 2 (*TCF7L2*), an established inducer of epithelial-to-mesenchymal transition and associated cancer invasion, was significantly upregulated in widely invasive tumors specifically (*p*<0.05; Fig. 1c).

### Immunohistochemistry

**Fig. 2:**
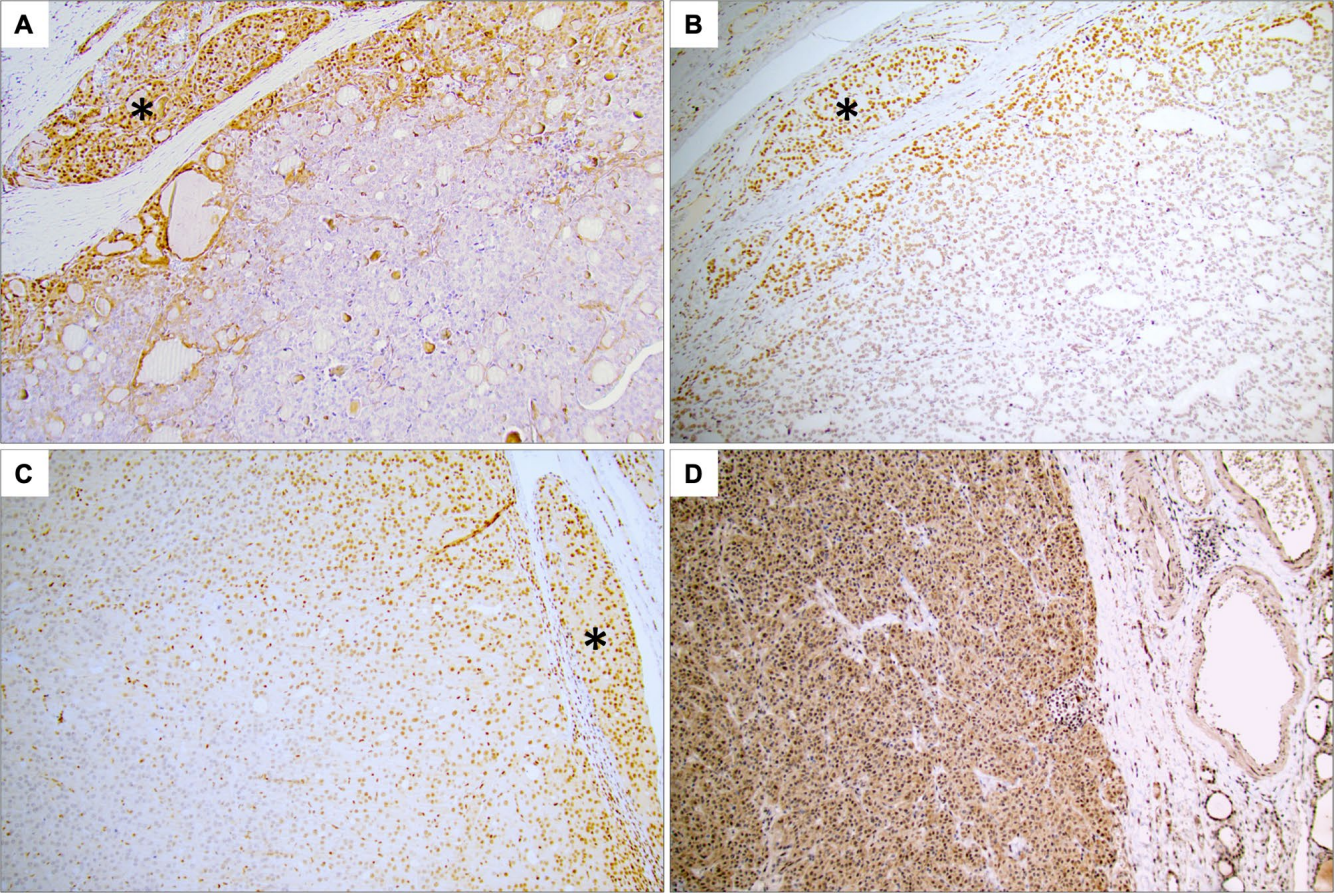
Protein expression of YY1 and MAX in follicular thyroid cancers and adenomas by immunohistochemistry.** a** YY1 expression in follicular thyroid carcinomas (FTCs) was specific to the invasive front, here exemplified by immunohistochemical staining of a widely invasive FTC (wiFTC). Note the increased nuclear staining intensity within the tumor cells adjacent to the capsule as well as in the intravascular tumor deposit (asterisk). **b** Additional wiFTC case displaying augmented YY1 expression at the invasive front (asterisk) compared with central parts of the tumor. **c** Additional FTC with vascular invasion (asterisk) stained for MAX. Note the intensified nuclear immunoreactivity in the intravascular tumor deposit compared with more central aspects of the tumor. **d** Nuclear and cytoplasmic MAX expression in a follicular thyroid adenoma, without apparent regional differences in immunoreactivity. All photomicrographs were magnified × 100

Immunostaining of candidate proteins MAX, TCF7L2, YY1, IRF1, STAT6, SP1, and E2F1 (selected based on qPCR results and/or associations to cancer in general) was performed in additional FTC specimens as outlined in the “Materials and Methods” section. For YY1, weak focal nuclear and cytoplasmic expression was generally observed in tumors, except for invasive fronts and intravascular foci—in which the nuclear staining was markedly increased in 17/19 informative tumors in which the stained section demonstrated identifiable invasive properties (Table 2; Fig. 2a–c). The same trend was noted for MAX, with increased nuclear expression in invasive areas in 13 out of the 16 FTCs in which a clear-cut corresponding invasive focus could be seen in the stained section (Table 2). YY1/MAX expression in the tumor bulk and surrounding normal tissue was variable without any particular correspondence with the invasive subtype of the tumor. In detail, for informative cases (section displaying an invasive focus), YY1 immunoreactivity was intensified across invasive foci compared with central tumor areas in 1/2 miFTCs, 4/4 in eaFTCs, and in 12/13 wiFTCs, and the same pattern for MAX was observed in 0/2 miFTCs, 2/3 eaFTCs, and in 11/13 wiFTCs (Table 2). To compare these findings with benign follicular thyroid tumors, we specifically looked for YY1 and MAX expressional differences in areas close to the capsule vs. central parts of the tumors in 10 FTAs. While all FTAs displayed medium levels of nuclear YY1 immunoreactivity, 6/10 FTAs displayed variable nuclear MAX expression while the remaining four samples exhibited a predominant cytosolic staining pattern with negative nuclear expression. Even so, all 10 cases stained homogenously for both markers when comparing central and capsule-near aspects in each individual tumor, thereby setting them apart from the FTC cohort (Fig. 2).

**Table 2:** Detailed results of the spatial YY1 and MAX immunoreactivity in follicular thyroid carcinoma

		YY1 nuclear immunoreactivity		MAX immunoreactivity	
FTC no.	Diagnosis (WHO 2017)	Central tumor component	Invasive focus	Central tumor component	Invasive focus
1	wiFTC	Negative	Strong	Negative	Weak
2	wiFTC	Negative	Strong	Weak	Medium
3	miFTC	Medium	N.i	Weak	N.i
4	eaiFTC	Negative	Strong	Weak	Medium
5	eaiFTC	Negative	Medium	Weak	Medium
6	eaiFTC	Negative	Strong	Weak	N.i
7	eaiFTC	Negative	Medium	Medium	Medium
8	wiFTC	Strong	Strong	Medium	N.i
9	wiFTC	Weak	Strong	Weak	N.i
10	miFTC	Strong	Strong	Medium	Medium
11	wiFTC	Negative	Medium	Weak	Medium
12	wiFTC	Negative	Strong	Weak	Medium
13	wiFTC	Negative	Medium	Weak	Medium
14	wiFTC	Negative	Strong	Weak	Medium
15	miFTC	Weak	Strong	Weak	Weak
16	wiFTC	Weak	Strong	Weak	Medium
17	wiFTC	Negative	Strong	Negative	Medium
18	wiFTC	Medium	Strong	Negative	Medium
19	wiFTC	Negative	Strong	Negative	Medium
20	wiFTC	Negative	Strong	Weak	Medium

*miFTC* minimally invasive follicular thyroid carcinoma, *eaFTC* encapsulated angioinvasive follicular thyroid carcinoma, *wiFTC* widely invasive follicular thyroid carcinoma, *N.i.* not informative (no invasive front on section)

STAT6 and IRF1 immunohistochemistry showed no apparent differences between central or invasive areas, thereby also serving as internal controls of adequate fixation. Other candidate markers with significantly altered mRNA levels from the qPCR array (SP1, E2F1, TCF7L2) exhibited comparable levels between tumor and adjacent normal tissues using immunohistochemistry without evident heterogeneity in terms of spatial distribution (data not shown).

**Fig. 3:**
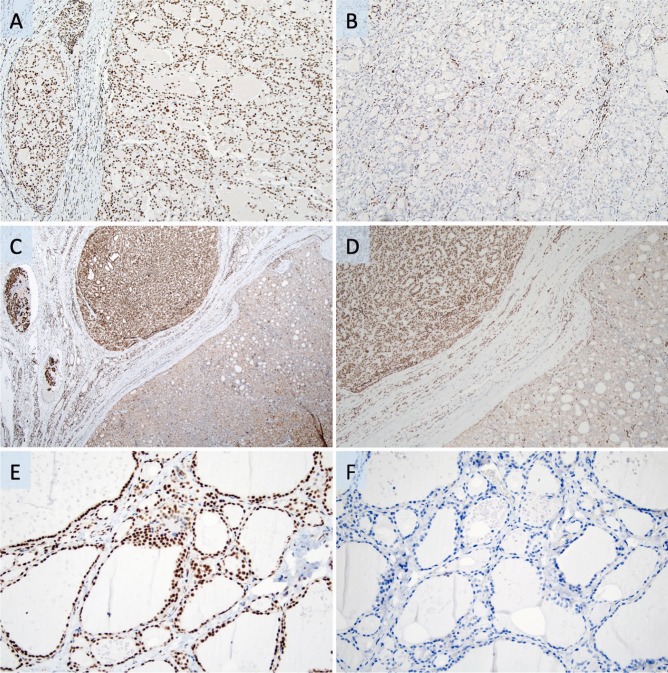
HDAC1 protein expression in the invasive fronts of follicular thyroid cancers. Photomicrographs depicting the heterogeneous HDAC1 immunoreactivity in follicular thyroid carcinoma (FTC). Magnification ×100 if not otherwise specified. **a** Area near the FTC capsule with an associated intravascular tumor deposit displaying strong nuclear HDAC1 immunoreactivity. **b** Same FTC specimen, illustrating negative or faintly weak nuclear staining in central aspects. Note the stromal cells acting as an internal positive control. **c** Separate FTC with several intravascular tumor deposits with intense HDAC1 nuclear staining (× 40 magnification), compared with weaker expression in tumor cells not engaging capsule or vessels. **d** Same case at × 100 magnification. **e** Normal thyroid epithelium as positive control. **f** Same normal thyroid tissue with the primary antibody omitted

Based on gene interaction analysis that implicated histone deacetylation as a potential mediator of the function of these aberrantly expressed transcription factors, immunohistochemistry was also performed for HDAC1 as well (Fig. 3a–f). The HDAC1 results were similar to those previously mentioned for the highlighted transcription factors, with higher expression along the invasive front than in the bulk of the tumor for 6 out of the 10 informative FTCs stained (2/4 miFTCs, 1/1 eaFTC and 3/5 wiFTCs). For the 10 FTAs, HDAC1 immunoreactivity was uniformly positive in all cases, with medium levels of nuclear immunoreactivity without regional differences in expression.

### Protein Interaction Analysis

**Fig. 4:**
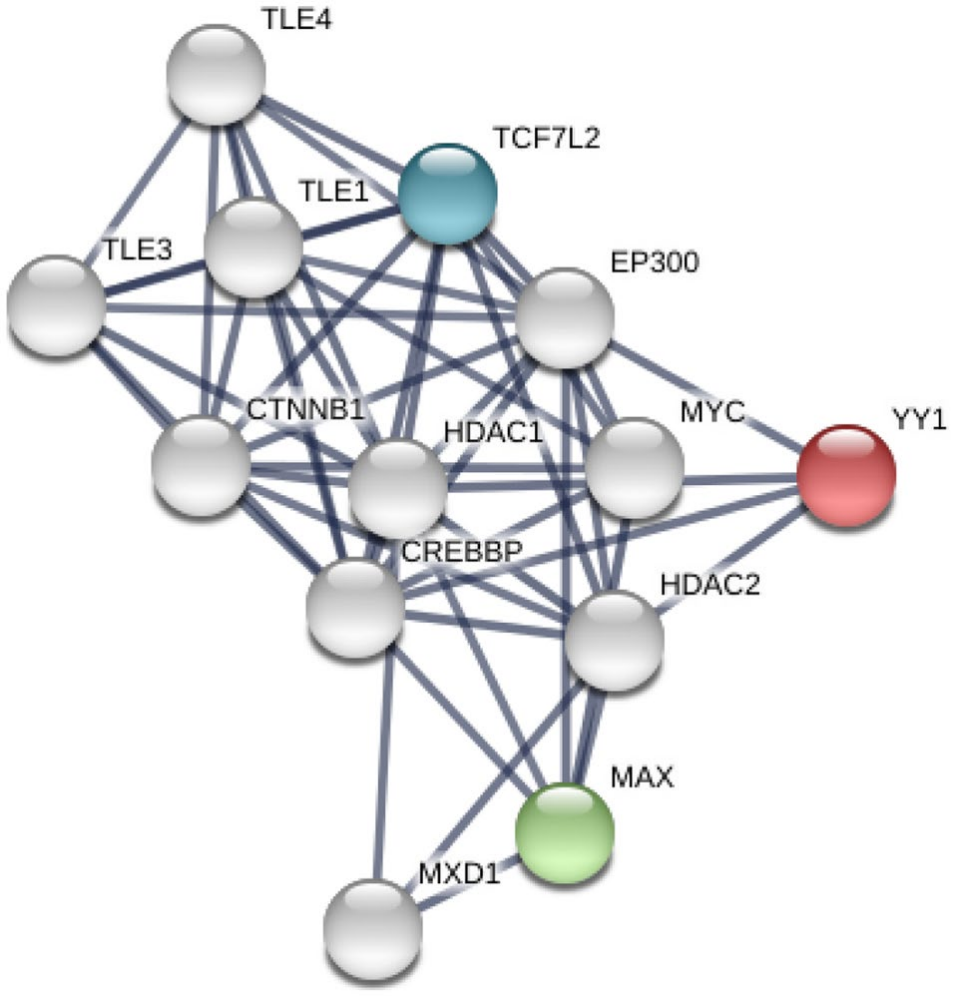
In silico protein-protein interaction network of putative invasion-mediating transcription factors in follicular thyroid cancer. Using *MAX*, *TCF7L2*, and* YY1* as the starting proteins, protein-protein interactions were mapped using the STRING tool. Histone modification was a recurring theme in the network, with *HDAC1*, *HDAC2*, and *EP300* all taking central roles

Based on the YY1 and MAX expression results via immunostaining, in silico protein-protein interaction mapping was performed. The STRING tool demonstrated numerous connections between the *MAX*, *YY1*, and *TCF7L2* proteins (Fig. 4). In particular, histone deacetylase enzymes 1 and 2 (*HDAC1*, *HDAC2*), as well as other histone acetylation modulators, seemed to be a common pathway associated with the expressed transcription factor proteins.

## Discussion

The diagnostic and therapeutic dilemma of how to manage indeterminate follicular thyroid lesions is a persistent challenge. FTCs are differentiated from follicular adenomas based on histological evaluation of the tumor’s invasive behavior, which cannot be determined using fine needle aspiration cytology. Further complicating the picture is the stark differences in patient outcome on the basis of the distinction between minimally invasive, encapsulated angioinvasive, and widely invasive FTC, again only evaluable on a resection specimen rather than needle aspirate [3, 6, 21].

Previous efforts to determine the genomic profile of invasive FTC relative to follicular adenoma or minimally invasive tumors have been largely unsuccessful, in part due to the heterogeneity of oncogenic drivers in follicular thyroid tumors [4, 8, 9]. A reliable marker for universal reflex screening is therefore not yet available for use in clinical practice, although some markers do show promising value in this context [15, 16]. In this background, our group performed expression profiling of selected transcription factors in a cohort of invasive FTC specimens, by qPCR and immunohistochemistry approaches, to investigate a potential invasion-promoting transcriptional switch. We found a few candidates for use as a reliable marker of the invasive phenotype, as *E2F1* gene output was increased in FTCs compared with normal thyroid samples, and increased expression of *TCF7L2* mRNA shows some promise as a marker for widely invasive tumors and would warrant further investigation in a larger cohort. However, these qPCR results were not reproduced on the protein level—as immunohistochemical analyses failed to see obvious differences in immunoreactivity in tumors compared with adjacent normal thyroid tissues. Our discordant findings might stem from the simple fact that mRNA and protein levels do not always exhibit a correlation in cellular systems, as differences in mRNA turnaround and regulatory mechanisms operating on the translational level might exist. Moreover, the risk of sampling bias should also be mentioned, as the tissue used for RNA extraction might not easily compare to the section used for immunohistochemistry, given the observably heterogeneous expression of transcription factors in general. Indeed, the lack of correlation was also the motivating force for us to screen for additional cancer-associated transcription factors using immunohistochemistry—even though the qPCR results indicated little or no apparent difference between tumor and normal tissues. In retrospect, the acquisition of RNA from FFPE would have allowed for a more direct comparison between RNA and protein levels on the same slide and could thus constitute an interesting follow-up study to our observations presented herein.

Speculatively, one of the reasons for the persistent challenges in identifying markers of invasive FTC may be genetic or epigenetic intra-tumoral spatial heterogeneity [22]. In support of this theory, we identified significant differences between protein expressions of some transcription factors in the bulk of the tumor when compared with the invasive front by immunohistochemistry. Notably, YY1 is coupled to invasive properties of malignant tumors, and the gene is in part regulated by *PTEN*—a tumor suppressor gene recurrently mutated and deleted in FTC [23–25]. On a similar note, *MAX* is an oncogene implicated in cell proliferation, differentiation, and apoptosis of certain tumor types [26]. Therefore, our findings of intensified YY1 and MAX protein expression at invasive areas makes reasonable biological sense—the cellular machinery in a cancer cell hard at work penetrating the tumor capsule might indeed be quite different from those pathways activated in a cell within the tumor bulk, which might be senescent, hypoxic, or actively proliferating, depending on the circumstances. However, these results pose further challenge to the usual diagnostic approach for thyroid tumors, which depends on needle aspiration of a random portion of thyroid nodule tissue rather than a sampling of any invasive portion specifically. Therefore, it may be that the bulk of the tumor of a miFTC and wiFTC is in fact indistinguishable on a genomic basis and may require histology to arrive at a definitive diagnosis. A recent study demonstrated that follicular adenoma and follicular carcinomas are possibly the same disease at different points on a spectrum, again questioning the possibility of a definitive diagnosis based on applied genomics from a needle aspiration [27]. The findings of our study may additionally provide some opportunity for a better classification of lesions which are not histologically widely invasive but express markers of invasion highly, which might direct more appropriate surgical or medical therapy for these high-risk patients. These speculations are supported by the finding of spatially homogenous YY1, MAX, and HDAC1 expressions in FTAs, strengthening our hypothesis that the visualized upregulation near invasive foci in FTCs are indeed a biologically relevant observation.

The invasive pathways identified in these regions of the tumors may represent attractive targets for therapy; however, common to the transcription factors identified in these regions seemed to be the modulation of histone acetylation via HDAC1 and other enzymes. Indeed, the histone deacetylase protein upregulation seemed to be specific to the invasive front of the tumors. Whether this represents a pathway specific to the invasion machinery itself or an adaptation to new non-tumorous surrounding tissue remains to be seen, but the availability of novel HDAC inhibitor drugs (currently in use for some hematologic malignancies) could present an intriguing possibility for patients with metastatic or even high-risk localized FTC, if these results can be further validated in vitro and in vivo. Surprisingly, a recent clinical trial evaluating one HDAC inhibitor for advanced thyroid cancers showed disappointing results, although the study was not performed in FTC specifically [28].

We conclude that the cancer-associated transcription factors YY1 and MAX exhibit expressional heterogeneity in FTCs. Strikingly, the nuclear expression of these proteins was augmented in the invasive fronts of the tumors compared with more central areas in the majority of informative cases—suggesting a potential role for these transcription factors in orchestrating the invasive behavior in FTCs. This hypothesis was strengthened by in silico analyses of YY1-MAX circuitry protein interactions, revealing direct associations to several histone modifiers—of which we confirmed similar intensified expression at invasive fronts for one of these: HDAC1. Overall, the spatially specific expression suggests that transcription factors linked to the epigenetic regulation of DNA accessibility might contribute to the invasive properties in FTCs. Moreover, our data suggest that a bulk genomic profiling approach for these tumors may not be sufficient for molecular diagnosis, given the spatial heterogeneity of these lesions. However, studying these invasive regions may provide insight into potentially targetable mechanisms for patients who require systemic therapies for refractory disease.

The data supporting the findings of this study are available within the article and its supplementary materials.

## Electronic supplementary material

Below is the link to the electronic supplementary material.

Supplementary file1 (DOCX 55.3 kb)
