# Supplementary material for: Transcription Factor Profiling Identifies Spatially Heterogenous Mediators of Follicular Thyroid Cancer Invasion
Source: Endocr Pathol. 2020 Oct 16;31(4):367–76. doi: 10.1007/s12022-020-09651-0 (PMC7666283; doi:10.1007/s12022-020-09651-0)
Supplement: Supplementary file 1 — Supplementary file1 (DOCX 55.3 kb) [file 12022_2020_9651_MOESM1_ESM.docx]

| **Supplementary Table 1. List of transcription factors included in the screening.** | | |
| --- | --- | --- |
| AR | HAND1 | POU2AF1 |
| ARNT | HAND2 | PPARA |
| ATF1 | HDAC1 | PPARG |
| ATF2 | HIF1A | RB1 |
| ATF3 | HNF1A | REL |
| ATF4 | HNF4A | RELA |
| CEBPA | HOXA5 | RELB |
| CEBPB | HSF1 | SMAD1 |
| CEBPG | ID1 | SMAD4 |
| CREB1 | IRF1 | SMAD5 |
| CREBBP | JUN | SMAD9 |
| CTNNB1 | JUNB | SP1 |
| DR1 | JUND | SP3 |
| E2F1 | MAX | STAT1 |
| E2F6 | MEF2A | STAT2 |
| EGR1 | MEF2C | STAT3 |
| ELK1 | MYB | STAT4 |
| ESR1 | MYC | STAT5A |
| ETS1 | MYF5 | STAT5B |
| ETS2 | MYOD1 | STAT6 |
| FOS | NFAT5 | TBP |
| FOXA2 | NFATC1 | TCF7L2 |
| FOXG1 | NFATC2 | TFAP2A |
| FOXO1 | NFATC3 | TGIF1 |
| GATA1 | NFATC4 | TP53 |
| GATA2 | NFKB1 | YY1 |
| GATA3 | NFYB |  |
| GTF2B | NR3C1 |  |
| GTF2F1 | PAX6 |  |
